# Pulmonary arterial compliance as a measure of right ventricular loading in mitral regurgitation

**DOI:** 10.1016/j.ijcha.2024.101472

**Published:** 2024-07-26

**Authors:** Hannah Kempton, Sara Hungerford, David W. Muller, Christopher S. Hayward

**Affiliations:** a Department of Cardiology, St Vincent’s Hospital, Sydney, Australia; b Faculty of Health and Medicine, The University of New South Wales, Sydney, Australia; c The CardioVascular Center, Tufts Medical Center, Boston MA, United States; d Department of Cardiology, Royal North Shore Hospital, Sydney, Australia; e Victor Chang Cardiac Research Institute, Sydney, Australia

**Keywords:** Pulmonary arterial compliance, Mitral regurgitation, Pulmonary hypertension, Right heart failure

## Abstract

Pulmonary hypertension (PH) is a common and prognostically important complication of mitral regurgitation (MR). Mean pulmonary arterial pressure (mPAP) and pulmonary vascular resistance (PVR) are traditionally used to diagnose PH, however these indices measure static rather than pulsatile load, leading to an incomplete representation of pulmonary vascular load on the right ventricle (RV). Pulmonary arterial compliance (PAC) is one method for quantifying pulsatile load, and is both a stronger predictor of prognosis in left heart failure, as well as a more sensitive measure of early pulmonary vascular dysfunction than PVR. With the expansion of transcatheter mitral and tricuspid valve therapies, there is renewed interest to more accurately define the load imposed by the pulmonary vasculature on the RV, especially in the early phase, prior to the onset of chronic PH. This review discusses the pathophysiology of pH in left heart failure and MR, the utility of PAC as a measure of RV afterload, and its calculation for clinical use and interpretation, underlining the utility of PAC as an adjunct for assessing pulmonary vascular haemodynamics.

## Graphical abstract

**Figure:**
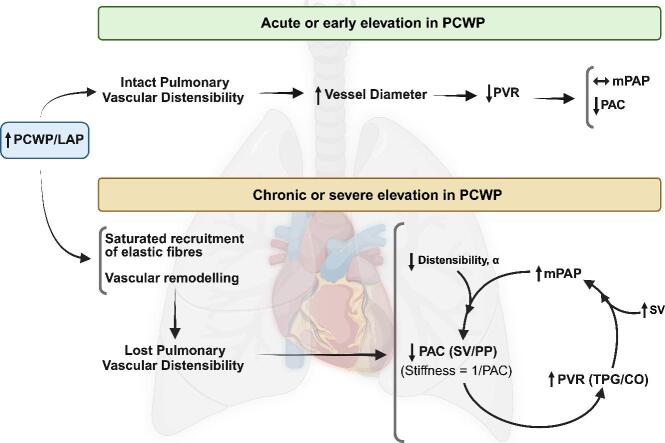

## 1 Background

Left heart failure is the most common cause of pulmonary hypertension (PH), complicating an estimated 60 to 70 % of cases [1], [2]. Left heart failure is frequently associated with valvular heart disease, and mitral regurgitation (MR) is the most commonly encountered valve lesion [3], in which the development of pH and right heart failure are important determinants of prognosis [4], [5], [6], [7], [8], [9], [10]. Co-existing PH is an important prognostic indicator across the spectrum of valvular heart disease and heart failure [11], [12], [13], Elevated pulmonary pressures have also been associated with poor prognosis following mitral transcatheter edge-to-edge repair (TEER), even despite a significant reduction in MR severity [14], [15], [16]. Accurate identification of the presence, severity and aetiology of pH is therefore important in the management of these patients.

There is some evidence that earlier treatment of MR is prognostically beneficial [17], [18], [19], [20], however for primary MR (PMR) guidelines currently recommend intervention for symptomatic severe MR, and watchful waiting for asymptomatic cases until onset of left ventricular (LV) impairment or dilatation, significant atrial dilatation, atrial fibrillation or pulmonary hypertension [21]. For secondary MR (SMR), the decision for intervention is also impacted by the underlying mechanism and indications for concurrent surgical intervention. Analyses of the results from the COAPT and MITRA-FR trials suggest a prognostic benefit for intervention in patients with MR severity in excess of LV dilatation, prior to the onset of LV decompensation [22], [23].

Therefore, central to the management and intervention for MR is the timely diagnosis of secondary complications of MR, especially in asymptomatic patients. Considering the prognostic significance of secondary PH, identification of those patients who are developing pulmonary vascular dysfunction so they may be considered for intervention, or at least more frequent monitoring, prior to the onset of frank PH is important. Traditionally, diagnosis of pH relies on pulmonary vascular resistance (PVR) and mean pulmonary arterial pressure (mPAP), however these parameters alone can be insensitive for detecting early increases in right ventricular (RV) afterload [8], and while PVR measures static pulmonary vascular load [24], it does not reflect the pulsatile load caused by wave reflections and vessel elasticity, which makes a significant contribution to RV afterload [25].

Pulsatile load and vascular elasticity are however reflected by pulmonary arterial compliance (PAC), which is more strongly associated with procedural outcomes, RV function and long-term prognosis in some cohorts than PVR [8], [26]. A decline in PAC is observed earlier in the course of pH than a rise in PVR, suggesting that PAC is also an earlier or more sensitive marker of elevated RV load [27]. This review discusses the pathophysiology of pH in left heart failure and MR, the clinical utility of PAC as an adjunct measure for assessing pulmonary vascular load on the RV, how it can be calculated clinically, and its potential application in MR and transcatheter therapies.

## 2 The pulmonary vascular response to mitral valve regurgitation and intervention

Unlike the systemic circulation, the pulmonary circulation is low pressure, and highly compliant, imposing a relatively low afterload on the RV, which demonstrates high capacity for changes in volume, but poor tolerance for elevated pressure [28].

Right heart catheterisation (RHC) in early severe MR shows normal pulmonary pressures, with the presence of a ‘V’ wave, depending on the compliance of the left atrium (LA) and pulmonary vasculature, corresponding to the transmission of left ventricular (LV) systolic pressure into the pulmonary vessels during systole. The role of LA compliance and function in the pathophysiology of pH is demonstrated in patients with both MR and mitral stenosis, and parameters including LA size, and LA strain assessments have been associated with mortality in both PMR and SMR [29], [30], [31], [32]. The pulsatile component of the LA pressure also appears to play a major role in reduced distensibility and hypertension in the pulmonary arteries in severe MR. Loss of LA compliance and the ability for this chamber to absorb the pulsatile load transmitted from the LV in MR is therefore likely to make an as yet unquantified contribution to the RV load in severe MR [33], [34].

With chronic severe MR, secondary left heart failure ensues, with a rise in LA pressure, and the onset of isolated post-capillary pulmonary hypertension (Ipc-PH), characterised by elevated pulmonary capillary wedge pressure (PCWP) (>15 mmHg) and mean pulmonary arterial pressure (mPAP) (>20 mmHg), with normal PVR (<2WU) [14], [35]. Chronically elevated PA pressures trigger adaptive changes in the pulmonary vasculature, initially with vasoconstriction, but ultimately vascular remodelling, which impairs vascular function, and increases PVR [36], [37], [38]. This results in combined pre- and post-capillary pulmonary hypertension (Cpc-PH), characterised by elevated PCWP, mPAP and PVR. Traditionally, PVR and the transpulmonary gradient (TPG) are used to distinguish Ipc-PH from Cpc-PH, with the latter considered the result of chronic pulmonary vascular remodelling in the face of chronically elevated pulmonary pressures [4], [37]. This is however an incomplete, and probably oversimplified description of the haemodynamic consequences of left heart failure and MR, as it omits the effects of pulsatile loading, which accounts for approximately 25 % of the RV afterload [8]. Further, cohorts of patients with severe MR and elevated pre-operative PVR can show dramatic improvement in mPAP, PVR and PCWP post-operatively, supporting the notion that even in the presence of Cpc-PH, a significant proportion of pulmonary vascular load may be reversible with correction of the valve lesion [39], [40]. In patients with pre-procedural RV dysfunction and MR, there is also frequently an improvement in RV function and reduction in the severity of tricuspid regurgitation (TR) following mitral TEER [41], [42], [43], [44], reflecting a reduction in RV afterload due to a fall in left atrial and pulmonary pressures, as well reverse pulmonary vascular remodelling, and improvements in RV function through ventricular interdependence, following relief of the volume load on the LV [45].

Conversely, in some cases PH and RV function do not necessarily improve after intervention, nor are pre-procedural mPAP and PVR necessarily predictive of post-procedural RV function [42]. After mitral TEER, approximately 20 % of patients have reduced RV function, associated with increased mortality, and this significant proportion of patients is not necessarily identified by elevated pulmonary arterial systolic pressure or RV dysfunction during pre-procedural assessment [42]. While this may reflect heterogeneity in patient selection, it may also reflect the insensitivity or shortcomings of echocardiographic assessment and PVR to diagnose patients with pre-procedural PH and RV dysfunction, PAC may therefore offer an additional, more sensitive measure to complement echocardiography and right heart catheterisation, to more completely characterise RV afterload.

## 3 Pulmonary arterial compliance

Compliance measures an important additional dimension of pulmonary vascular function, and can be considered as the third component of the triad of pressure, resistance and compliance that can describe RV afterload (Central Illustration). Compliance represents the elastic properties of the pulmonary vascular system, and is defined by the change in volume per unit change in pressure [46]. The PAC is proportional to the stiffness of the arterial wall, and this relationship is governed by elasticity, such that as arterial pressure increases, and the elasticity of the arterial wall becomes saturated, the arterial wall stiffness increases, and compliance falls. Compliance is therefore dependent on pressure, but will also be reduced by pulmonary vascular remodelling, as elastic fibres are replaced by collagen and fibrosis [47]. PAC is just one of a number of methods that give a clinical approximation of pulsatile load. While some methods, for example pulmonary vascular impedance, give a more comprehensive description of the components of pulmonary vascular mechanics, the clinical acquisition and interpretation of such data is cumbersome and therefore usually clinically prohibitive, making PAC and related models of RV loading, obtained during RHC, more clinically viable [48], [49].

The effect of MR on PAC is not well described, however, similar to left heart failure cohorts, MR increases LA and pulmonary arterial pressure, causing stretch of the elastic fibres in the pulmonary arteries, subsequently reducing PAC. As a dynamic and early haemodynamic consequence of this process, a reduction in PAC is therefore more sensitive than PVR for detecting early and acute changes in pulmonary vascular congestion and RV afterload, prior to the onset of pulmonary vascular remodelling.

Despite being a key physiological parameter that gives an additional dimension to the description of pulmonary vascular function and RV afterload, compliance is excluded from routine clinical assessment. This is due to both the lack of consensus for how it is measured, a lack of data that describes normal values, and to date, no strong clinical indication for an additional descriptor of pulmonary load above mPAP and PVR. However, in contrast to surgical interventions, where the heart is supported through these altered loading conditions with gradual weaning from cardiopulmonary bypass, with pharmacological supports, in transcatheter intervention the beating heart must tolerate instantaneous and significant changes in afterload. The rise of transcatheter therapies has therefore highlighted the importance of the accurate pre-operative diagnosis of ventricular function and loading. The prognostic significance of PAC in PH, as well as its ability to detect earlier pulmonary vascular dysfunction, and therefore potentially trigger decisions for earlier intervention, supports the addition of PAC to the pre-procedural assessment of pH and RV function in patients with MR [25], [28], [50], [51].

## 4 Pulmonary vascular resistance and mean pulmonary arterial pressure

PVR is calculated from the transpulmonary gradient (TPG), the difference between the mPAP and PCWP, as a proportion of cardiac output, and it follows that at unchanged CO, and resistive vessel diameter, mPAP increases proportionally to PCWP [52]. However, healthy pulmonary vessels are highly distensible, and as PCWP rises, the vessels distend, and the PCWP is transmitted in a less than one-to-one ratio (i.e. a 1 mmHg rise in PCWP results in a < 1 mmHg rise in mPAP). A vessel distensibility co-efficient can be incorporated into the PVR equation to account for this distensibility [53]. This coefficient has a normal value of 1–2 %/mmHg [52], [54], however, as could be expected, as the PCWP and mPAP rise, and vessel elasticity becomes saturated, the distensibility coefficient falls. This is associated with impaired RV function and survival [55]. Vessel distensibility reflects arterial stiffness, which is the inverse of PAC, and increases with increasing pulmonary arterial pressure [56]. Pulmonary arterial pressure is strongly and positively correlated with arterial stiffness, and the relationship between resistance, pressure and compliance is such that increased PVR raises the mPAP, thereby reducing the PAC [49], [57].

In left heart failure, the left ventricular end diastolic and LA pressures rise, with transmission of pressure into the pulmonary vascular system, reflected in the elevated mPAP. In a system where pulmonary vascular distensibility is intact, as luminal pressure rises acutely, elastic fibres in the arterial walls are recruited and the vessels dilate, thereby initially reducing both the magnitude of the pressure increase and the PVR. Vessel distensibility is however limited, and as the pressure continues to rise, this compensatory mechanism becomes saturated, and the pressure is transmitted directly from PCWP to mPAP [49]. This is highlighted in patients with left heart failure during exercise, where the slope of the mPAP/CO relationship is steeper than predicted by the PVR, owing in part to the increase PCWP, and hence mPAP, in the setting of impaired vessel distensibility [58].

If the pressure is relieved, elasticity of the elastic fibres can be restored. However, chronically elevated pressure causes vascular remodelling, with arterial stiffening due to collagen deposition and fibrosis, resulting in a reduction in the proportion of elastic fibres [59].

A disproportionate elevation in the pulmonary pressure gradient signifies the onset of Cpc-PH, defined by an elevated TPG, which reflects loss of pulmonary distensibility either due to saturation of elasticity, or fibrosis and remodelling [38], [60].

**Fig. 1:**
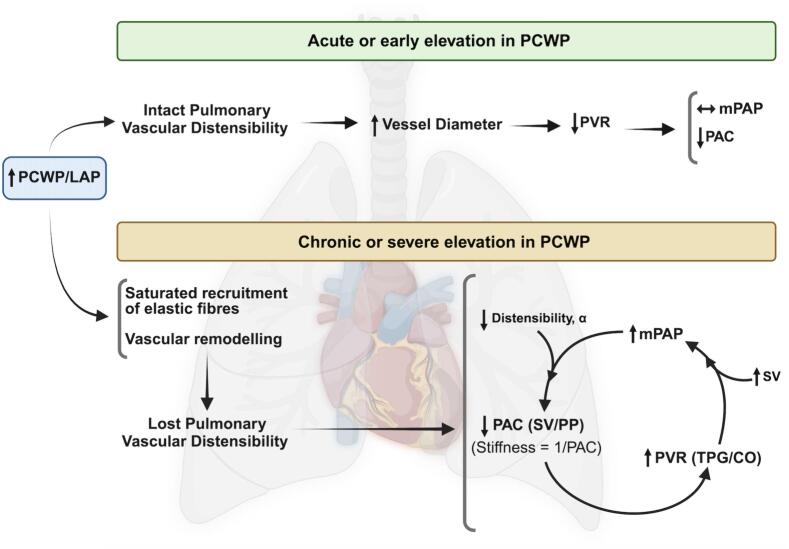
**Graphical Abstract** The interaction between pulmonary vascular resistance (PVR), pulmonary arterial pressure, and pulmonary arterial compliance in states of acute and chronically elevated left atrial pressure. α: arterial stiffness; CO: cardiac output; LAP: left atrial pressure; mPAP: mean pulmonary arterial pressure; PAC: pulmonary arterial compliance; PCWP: pulmonary capillary wedge pressure; PP: pulmonary arterial pulse pressure; PVR: pulmonary vascular resistance; SV: stroke volume; TPG: transpulmonary gradient. Created with BioRender.com.

To summarise, the compliance of the pulmonary vasculature can be impaired by two mechanisms. By an increase in pressure, which saturates the elasticity of the arterial elastic fibres, and the other by remodelling, which is considered less reversible, with increased vessel stiffness. These mechanisms co-exist, and provide one explanation for why even with Cpc-PH, reduction in PCWP, and therefore mPAP, can reduce the TPG and PVR. The interactions between PVR, mPAP and PAC are summarised in the graphical abstract (Fig. 1).

## 5 The resistance-compliance relationship

Unlike the systemic circulation, where compliance is mainly confined to the proximal aorta, and resistance to the distal arterioles and capillaries, in the pulmonary circulation there is no such anatomical separation, with resistance and compliance arising concurrently throughout the vascular bed [61]. This provides the anatomical basis for the resistance-compliance (RC) relationship, which is an inverse hyperbolic relationship that has been described between PAC and PVR in the pulmonary circulation[63], [64], and implies that the product of resistance ($\mathrm{mmHg}∙s∙{\mathrm{mL}}^{-1})$ and compliance ($\mathrm{mL}∙{\mathrm{mmHg}}^{-1})$ is a constant, the unit for which is time (RC time) [26], [33], [62], [65]. RC time is therefore an integrative measure that encompasses both compliance and resistance to represent total RV loading. and this forms the basis for what is termed the two-element Windkessel model [62].

The RC relationship implies that in early PH, small increases in PVR are accompanied by large reductions in PAC, and reductions in PAC have been observed in early PH, prior to any appreciable increase in PVR. Conversely, at high PVR (>3–4 WU), there is little incremental change in PAC, which, as could be expected, becomes maximally depressed in highly resistant, fibrosed and non-distensible pulmonary arteries [26]. In post-capillary PH, the RC relationship it is modified by elevated LA pressure, such that PAC is linearly reduced as LA pressure rises [33]. In other words, elevated PCWP lowers PAC, and therefore RC time. As could be expected, the converse is also true, in that as the PCWP is reduced, PAC improves, and the RC time increases [26]. This is in keeping with that described above, where, as the pulmonary arterial pressure falls, and the elasticity of the fibres in the arterial wall is restored, compliance improves.

The RC time can be defined as the time it takes for the PA pressure to drop by 63 % in diastole, and can therefore be obtained by pulmonary waveform analysis [46], [48]. Further, where RC time and PVR are known, and decay is mono-exponential, the PAC can be calculated.

Calculation of PAC from the RC time does however rely on the constancy of the inverse hyperbolic relationship between PAC and PVR, and while this has been demonstrated in multiple pre-capillary pulmonary hypertension cohorts [62], [66], [67], the constancy of the RC relationship has been questioned, especially in left heart failure, due to significant variability of the RC time with mPAP, heart rate and PCWP [56].

Further, the constancy of the inverse hyperbolic relationship between PAC and PVR may be artefactual. This relationship has been observed in cohorts in which the PVR is calculated by:

$$\mathrm{PVR}=\frac{\mathrm{mPAP}-PCWP}{\mathrm{SV}∙HR}$$

Where mPAP is the pulmonary arterial pressure, PCWP is the pulmonary capillary wedge pressure, SV is stroke volume, and HR is heart rate.

And PAC, calculated by the empiric formula:

$$\mathrm{PAC}=\frac{\mathrm{SV}}{\mathrm{PP}}$$

Where SV is stroke volume and HR is heart rate.

These equations are intrinsically linked by stroke volume, and the correlation of mPAP with the pulse pressure in an approximately 1:1 ratio. Both are also dependent on flow induced vascular recruitment and distensibility, which are appreciably affected by PCWP [56]. It can therefore be theorised that the observed constancy of this relationship is mathematical rather than physiological, and some authors therefore promote the use of isobaric PAC (PAC at a fixed mPAP) as a measure of compliance, rather than PAC based on PVR, for the quantification of RV afterload [56]. Regardless, this introduces a possible limitation in the ability to accurately ascertain PAC from RC time, and while RC time gives a useful integrative measure of RV afterload, encompassing both PVR and PAC, the assumption that accurate estimations of these components can be obtained on the basis of the inverse hyperbolic relationship is questioned.

Further, in the pulmonary circulation, pressure decays to the outflow pressure rather than zero. The outflow pressure is usually approximated by the PCWP, and where this is elevated, the PVR can therefore be underestimated. Similarly, in cases of pH where the outflow pressure exceeds the PCWP, for example in primary PH or Cpc-PH, the PVR can be overestimated, introducing additional error to the subsequent calculation of PAC [49].

Therefore, although RC time gives an integrative measure of RV load, the constancy in the relationship between PAC and PVR, is not sufficiently robust to reliably describe the individual components of RV load, especially where the PCWP is elevated, including in left heart failure and MR. As outlined below, there are multiple methods to measure RC time, however these are clinically cumbersome, and therefore, an understanding of the physiological components of RV loading, including pressure, compliance and resistance, and how these interact, achieves a more comprehensive understanding of pulmonary vascular function and RV load, from basic physiological principles, as summarised in Fig. 1 and described below.

## 6 Measurement of RC time and pulmonary arterial compliance

While multiple methods for measuring PAC have been described, the most clinically feasible, widely used, and well validated is the empiric method, the ratio of stroke volume (SV) to PA pulse pressure (PP), which can be obtained during RHC. Despite common use, the SV/PP ratio overestimates PAC as the SV exceeds the reservoir volume of the pulmonary vascular system, and therefore does not account for flow from the pulmonary vascular bed, overestimating SV [46]. This estimation of PAC does however correlate with models of pulmonary impedance, and with outcomes in advanced heart failure [26], [68]. It also allows calculation of PAC from directly measured haemodynamic parameters, reducing the potential for error and artefact. Alternative methods for calculating PAC are based on its derivation from RC time, and include the diastolic pressure decay, area under the curve, semi-logarithmic, and logarithmic pressure difference methods, each of which are presented here.

The diastolic pressure decay method is based on the premise that in a Windkessel model, the pressure drops mono-exponentially after closure of the pulmonary valve, according to [69]:

$$P\left(t\right)=P_{\mathrm{diast}}e^{-t/RC}$$

Fitting the diastolic pressure curve to an exponential curve gives the RC time, from which the compliance can be calculated if the PVR is known.

The area method uses SV and the area under the pressure curve in systole and diastole to calculate compliance [70], according to:

$$\mathrm{PAC}=\frac{\mathrm{SV}}{K(P_{\mathrm{earlydiastole}}-P_{\mathrm{enddiastole})}}$$

Where $K=\frac{A_{s}-A_{d}}{A_{d}}$, and A is the area in systole and diastole

The semi-logarithmic method calculates RC time by [49]:

$$\mathrm{RC}=\frac{\mathrm{diastolictime}}{ln(P_{0}-P_{\mathrm{asymptote}}/(dPAP-P_{\mathrm{asymptote}))}}$$

Where LVEDP or PCWP is used as P_asymptote_

The logarithmic pressure difference method gives a measure of PAC, independent of the RC time, and calculation from pressure curves [66]:

$$\mathrm{PAC}=\frac{t}{\mathrm{PVR}∙\log_{e}\frac{\left(P_{0}-{\mathrm{LA}}_{P}\right)}{{(P}_{t}-{\mathrm{LA}}_{P})}}$$

Where P_0_ is the pulmonary arterial pressure at the dicrotic notch, P_t_ is pulmonary arterial pressure at the end of diastole, t is time between P_0_ and P_t_, and LA_p_ is left atrial pressure, taken as PCWP for most purposes [66].

Finally, Lankhaar derived an empirical equation, based on RC time [62]:

$$\tau =RC=\frac{\mathrm{mPAP}-PCWP}{\mathrm{SV}/T}\times \frac{\mathrm{SV}}{\mathrm{PP}}=T\frac{\mathrm{mPAP}-PCWP}{\mathrm{PP}}$$

Where τ is the RC time, and T is the heart period.

While each of these methods is acceptable, many have been adapted from studies examining compliance in the systemic rather than the pulmonary circulation. Further, while it has been suggested that they may provide a more accurate measure of PAC, clinically they are not as straight-forward to obtain as SV/PP, and, except for the logarithmic pressure difference method, all require specialist software for wave acquisition and transformation. In pulmonary hypertension cohorts, SV/PP is the most widely used, and validated, with data to support the prognostic utility of this method. Finally, and perhaps most significantly, excepting the logarithmic pressure difference and area under the curve methods, these methods derive the PAC from the RC time and PVR, which, as discussed above, is likely not sufficiently robust to give an accurate estimation of PAC, especially in cases where the PCWP is elevated. Further exploration of the relationship between PAC and PVR under different pathological conditions is warranted to evaluate the ability to derive PAC from RC time, and accurately break RC time, or the 2-element Windkessel model, into its constituent parts of PVR and PAC.

Finally, a non-invasive, echocardiographic method for measuring PAC has also been described, and validated against PAC measured by SV/PP, in which the RV outflow tract velocity–time integral (RV VTI) is taken as a ratio of PASP [71]:

$$\mathrm{PAC}=\frac{{\mathrm{RVOT}}_{\mathrm{VTI}}}{\mathrm{PASP}}$$

Where RVOT_VTI_ is the velocity–time integral in the RVOT and PASP is the pulmonary arterial systolic pressure.

While as an echocardiographic measure, it is more prone to error due to image quality, and Doppler angulation, the advantage of a non-invasive method for obtaining PAC lies in the capacity for serial measurements. It has also been associated with mortality in PH [72], [73].

## 7 Clinical implications

As it stands, elevated PVR and mPAP identify significant PH in patients with MR. However, as has been discussed in this review, pressure and resistance become elevated only once pressure in the pulmonary vasculature is high enough to have saturated the elasticity of the pulmonary arteries, and/or chronically elevated pressure has resulted in stiffening through fibrosis and remodelling of the pulmonary vascular bed. Persistent PH after mitral valve intervention contributes to ongoing morbidity and higher mortality, and a shift to promoting earlier valve repair, prior to the onset of pH may be beneficial [74]. With changes detectable prior to the onset of raised mPAP or PVR, there is therefore a role for PAC in the assessment of patients with MR. By virtue of the mechanics of the pulmonary vascular system, reduced PAC is an early marker of increasing RV load and could therefore be used to identify patients with early pulmonary vascular dysfunction who should be more closely monitored, or perhaps, in consideration with other factors, referred for earlier intervention. Certainly, PAC can be taken from RHC, without a need for additional investigations, which makes it a viable additive parameter to complete the triad of pressure, compliance and resistance the describe pulmonary vascular haemodynamics.

Another clinical application of PAC may be to assess RV loading in TR. Of particular interest is the identification of methods to determine RV loading prior to transcatheter tricuspid valve intervention, with the aim of diagnosing the RV that is unlikely to manage the acute elevation in afterload associated with the cessation of severe TR. Similar to MR, invasive haemodynamic parameters, including mPAP and PVR predict survival following tricuspid TEER [75], and PAC may also be additive in this context, where more sensitive prediction of pulmonary vascular dysfunction may identify at-risk ventricles prior to intervention. Similarly, in those patients with both TR and MR, PAC presents an opportunity to characterise more subtle pulmonary vascular dysfunction, that may contribute to understanding the interaction between these valve lesions, and the prognosis for TR following mitral valve intervention.

What remains unknown is the value for PAC that should be considered abnormal. A recent study [51] identified PAC<3.0 mL/mmHg as prognostically significant for survival in patients with PH, including with left heart failure, and cohort validation studies are required to understand this in the setting of MR.

## 8 Conclusion

Pulmonary hypertension is a common and prognostically important complication of MR, where pressure from the left heart is transmitted to the pulmonary vasculature, increasing the RV afterload. Traditionally, PH in left heart failure is defined by the PCWP, mPAP, and PVR, however while these parameters are fundamental, a measure of elastic potential or compliance is required to obtain a complete picture of pulmonary haemodynamic function. PAC provides a measure of this pulsatility, and further, is more sensitive for early pulmonary vascular dysfunction than either mPAP or PVR, which can remain normal in the early phases of severe MR. The use of PAC as an adjunct measure during RHC therefore presents an opportunity to diagnose early MR-associated pulmonary vascular dysfunction, when intervention prior to the onset of pulmonary vascular remodelling is most desirable, however methods to accurately quantify PAC, especially in the context of severe MR, require further elucidation.

**Disclosures:** The authors have no disclosures.

## CRediT authorship contribution statement

**Hannah Kempton:** Writing – review & editing, Writing – original draft, Project administration, Conceptualization. **Sara Hungerford:** Writing – review & editing, Supervision, Conceptualization. **David W. Muller:** Writing – review & editing, Supervision, Conceptualization. **Christopher S. Hayward:** Writing – review & editing, Supervision, Conceptualization.

## Declaration of competing interest

The authors declare that they have no known competing financial interests or personal relationships that could have appeared to influence the work reported in this paper.

## References

[1] Hoeper M.M., Humbert M., Souza R., Idrees M., Kawut S.M., Sliwa-Hahnle K., Jing Z.-C., Gibbs J.S.R. (2016). A global view of pulmonary hypertension. Lancet Respir. Med..

[2] Tichelbäcker T., Dumitrescu D., Gerhardt F., Stern D., Wissmüller M., Adam M., Schmidt T., Frerker C., Pfister R., Halbach M. (2019). Pulmonary hypertension and valvular heart disease. Herz.

[3] Vesely M.R., Benitez R.M., Robinson S.W., Collins J.A., Dawood M.Y., Gammie J.S. (2015). Surgical and Transcatheter Mitral Valve Repair for Severe Chronic Mitral Regurgitation: A Review of Clinical Indications and Patient Assessment. J. Am. Heart Assoc..

[4] Patel H., Desai M., Tuzcu E.M., Griffin B., Kapadia S. (2014). Pulmonary hypertension in mitral regurgitation. J. Am. Heart Assoc..

[5] Wencker D., Borer J.S., Hochreiter C., Devereux R.B., Roman M.J., Kligfield P., Supino P., Krieger K., Isom O.W. (2000). Preoperative Predictors of Late Postoperative Outcome among Patients with Nonischemic Mitral Regurgitation with 'High Risk' Descriptors and Comparison with Unoperated Patients. Cardiology.

[6] Kaneko H., Neuss M., Weissenborn J., Butter C. (2016). Prognostic Significance of Right Ventricular Dysfunction in Patients With Functional Mitral Regurgitation Undergoing MitraClip. Am. J. Cardiol..

[7] Giannini C., Fiorelli F., Colombo A., De Carlo M., Weisz S.H., Agricola E., Godino C., Castriota F., Golino P., Petronio A.S. (2016). Right ventricular evaluation to improve survival outcome in patients with severe functional mitral regurgitation and advanced heart failure undergoing MitraClip therapy. Int. J. Cardiol..

[8] Ghio S., Schirinzi S., Pica S. (2015). Pulmonary arterial compliance: How and why should we measure it?. Global Cardiol. Sci. Pract..

[9] van Wijngaarden A.L., Mantegazza V., Hiemstra Y.L., Volpato V., van der Bijl P., Pepi M., Palmen M., Delgado V., Ajmone Marsan N., Tamborini G. (2022). Prognostic Impact of Extra-Mitral Valve Cardiac Involvement in Patients With Primary Mitral Regurgitation. J. Am. Coll. Cardiol. Img..

[10] Doldi P.M., Stolz L., Kassar M., Kalbacher D., Petronio A.S., Butter C., von Bardeleben R.S., Iliadis C., Grayburn P., Hausleiter J. (2024). Paradox of disproportionate atrial functional mitral regurgitation and survival after transcatheter edge-to-edge repair. ESC Heart Failure..

[11] Henrique Rangel R., Christoph Voran J., Seoudy H., Villinger T., Lutter G., Puehler T., Kreidel F., Frank J., Salem M., Frank D. (2024). Transcatheter aortic valve replacement in patients with severe aortic valve stenosis and concomitant mitral valve regurgitation – 5 years follow-up. IJC Heart Vasc..

[12] Macera F., Dewachter C., Stefanidis C., Vanden Eynden F., Bondue A., Vachiéry J.-L., Roussoulières A. (2023). Lung diffusion capacity correlates with pre-implant pulmonary hypertension and predicts outcome after LVAD implantation. ESC Heart Failure..

[13] Viva T., Postolache A., Nguyen Trung M.-L., Danthine P., Petitjean H., Bruno V.D., Martinez C., Lempereur M., Guazzi M., Aghezzaf S. (2023). A new integrative approach combining right heart catheterization and echocardiography to stage aortic stenosis-related cardiac damage. Front. Cardiovascul. Med..

[14] Opotowsky A.R., Dimopoulos K., Buber J. (2020). Implications of Elevated Pulmonary Artery Pressure for Transcatheter Mitral Repair: Time for Comprehensive Hemodynamic Investigation∗. J. Am. Coll. Cardiol..

[15] Alessandro M-M, Lorenzo T, Luca A, Andrea D, Alberto S, Georgios T, Gabriele C, Emmanuel A, Alexandros B, Stefano Cornara KMKPhKMvr. Transcatheter mitral valve repair with MitraClip in patients with pulmonary hypertension: hemodynamic and prognostic perspectives. *Rev. Cardiovasc. Med.* 2021;22:33. 10.31083/j.rcm.2021.01.214 33792246

[16] Ben-Yehuda O., Shahim B., Chen S., Liu M., Redfors B., Hahn R.T., Asch F.M., Weissman N.J., Medvedofsky D., Puri R. (2020). Pulmonary Hypertension in Transcatheter Mitral Valve Repair for Secondary Mitral Regurgitation: The COAPT Trial. J. Am. Coll. Cardiol..

[17] Suri R.M., Vanoverschelde J.-L., Grigioni F., Schaff H.V., Tribouilloy C., Avierinos J.-F., Barbieri A., Pasquet A., Huebner M., Rusinaru D. (2013). Association Between Early Surgical Intervention vs Watchful Waiting and Outcomes for Mitral Regurgitation Due to Flail Mitral Valve Leaflets. J. Am. Med. Assoc..

[18] Montant P., Chenot F., Robert A., Vancraeynest D., Pasquet A., Gerber B., Noirhomme P., El Khoury G., Vanoverschelde J.-L. (2009). Long-term survival in asymptomatic patients with severe degenerative mitral regurgitation: A propensity score–based comparison between an early surgical strategy and a conservative treatment approach. J. Thorac. Cardiovasc. Surg..

[19] Kang D.-H., Kim J.H., Rim J.H., Kim M.-J., Yun S.-C., Song J.-M., Song H., Choi K.-J., Song J.-K., Lee J.-W. (2009). Comparison of Early Surgery Versus Conventional Treatment in Asymptomatic Severe Mitral Regurgitation. Circulation.

[20] Kang D.-H., Park S.-J., Sun B.J., Cho E.J., Kim D.-H., Yun S.-C., Song J.-M., Park S.W., Chung C.-H., Song J.-K. (2014). Early Surgery Versus Conventional Treatment for Asymptomatic Severe Mitral Regurgitation: A Propensity Analysis. J. Am. Coll. Cardiol..

[21] Vahanian A., Beyersdorf F., Praz F., Milojevic M., Baldus S., Bauersachs J., Capodanno D., Conradi L., De Bonis M., De Paulis R. (2022). 2021 ESC/EACTS Guidelines for the management of valvular heart disease: Developed by the Task Force for the management of valvular heart disease of the European Society of Cardiology (ESC) and the European Association for Cardio-Thoracic Surgery (EACTS). Eur. Heart J..

[22] Stone G.W., Lindenfeld J., Abraham W.T., Kar S., Lim D.S., Mishell J.M., Whisenant B., Grayburn P.A., Rinaldi M., Kapadia S.R. (2018). Transcatheter Mitral-Valve Repair in Patients with Heart Failure. N. Engl. J. Med..

[23] Obadia J.-F., Messika-Zeitoun D., Leurent G., Iung B., Bonnet G., Piriou N., Lefèvre T., Piot C., Rouleau F., Carrié D. (2018). Percutaneous Repair or Medical Treatment for Secondary Mitral Regurgitation. N. Engl. J. Med..

[24] Thenappan T., Prins K.W., Pritzker M.R., Scandurra J., Volmers K., Weir E.K. (2016). The Critical Role of Pulmonary Arterial Compliance in Pulmonary Hypertension. Ann. Am. Thorac. Soc..

[25] Vonk-Noordegraaf A., Haddad F., Chin K.M., Forfia P.R., Kawut S.M., Lumens J., Naeije R., Newman J., Oudiz R.J., Provencher S. (2013). Right Heart Adaptation to Pulmonary Arterial Hypertension: Physiology and Pathobiology. J. Am. Coll. Cardiol..

[26] Dupont M., Mullens W., Skouri H.N., Abrahams Z., Wu Y., Taylor D.O., Starling R.C., Tang W.H.W. (2012). Prognostic role of pulmonary arterial capacitance in advanced heart failure. Circ. Heart Fail..

[27] Tampakakis E, Shah SJ, Borlaug BA, Leary PJ, Patel HH, Miller WL, Kelemen BW, Houston BA, Kolb TM, Damico R, et al. Pulmonary Effective Arterial Elastance as a Measure of Right Ventricular Afterload and Its Prognostic Value in Pulmonary Hypertension Due to Left Heart Disease. *Circulation: Heart Failure*. 2018;11:e004436. 10.1161/CIRCHEARTFAILURE.117.004436 PMC5901761 29643065

[28] Konstam M.A., Kiernan M.S., Bernstein D., Bozkurt B., Jacob M., Kapur N.K., Kociol R.D., Lewis E.F., Mehra M.R., Pagani F.D. (2018). Evaluation and Management of Right-Sided Heart Failure: A Scientific Statement From the American Heart Association. Circulation.

[29] Ha J.-W., Chung N., Jang Y., Kang W.-C., Kang S.-M., Rim S.-J., Shim W.-H., Cho S.-Y., Kim S.S. (2000). Is the left atrial v wave the determinant of peak pulmonary artery pressure in patients with pure mitral stenosis?. Am. J. Cardiol..

[30] Stassen J., Namazi F., van der Bijl P., van Wijngaarden S.E., Kamperidis V., Marsan N.A., Delgado V., Bax J.J. (2022). Left Atrial Reservoir Function and Outcomes in Secondary Mitral Regurgitation. J. Am. Soc. Echocardiogr..

[31] Stassen J., van Wijngaarden A.L., Butcher S.C., Palmen M., Herbots L., Bax J.J., Delgado V., Ajmone M.N. (2023). Prognostic value of left atrial reservoir function in patients with severe primary mitral regurgitation undergoing mitral valve repair. Europ. Heart J. – Cardiovascul. Imag..

[32] Inciardi R.M., Rossi A., Bergamini C., Benfari G., Maffeis C., Greco C., Drago A., Guazzi M., Ribichini F.L., Cicoira M. (2020). Mitral regurgitation, left atrial structural and functional remodelling and the effect on pulmonary haemodynamics. Eur. J. Heart Fail..

[33] Tedford R.J., Hassoun P.M., Mathai S.C., Girgis R.E., Russell S.D., Thiemann D.R., Cingolani O.H., Mudd J.O., Borlaug B.A., Redfield M.M. (2012). Pulmonary Capillary Wedge Pressure Augments Right Ventricular Pulsatile Loading. Circulation.

[34] Najjar E., Lund L.H., Hage C., Nagy A.I., Johnson J., Manouras A. (2021). The Differential Impact of the Left Atrial Pressure Components on Pulmonary Arterial Compliance-Resistance Relationship in Heart Failure. J. Card. Fail..

[35] Humbert M, Kovacs G, Hoeper MM, Badagliacca R, Berger RMF, Brida M, Carlsen J, Coats AJS, Escribano-Subias P, Ferrari P, et al. 2022 ESC/ERS Guidelines for the diagnosis and treatment of pulmonary hypertension: Developed by the task force for the diagnosis and treatment of pulmonary hypertension of the European Society of Cardiology (ESC) and the European Respiratory Society (ERS). Endorsed by the International Society for Heart and Lung Transplantation (ISHLT) and the European Reference Network on rare respiratory diseases (ERN-LUNG). *Europ. Heart J*. 2022;43:3618-3731.

[36] Heath D., Edwards J.E. (1958). The Pathology of Hypertensive Pulmonary Vascular Disease. Circulation.

[37] Allen B.J., Frye H., Ramanathan R., Caggiano L.R., Tabima D.M., Chesler N.C., Philip J.L. (2023). Biomechanical and Mechanobiological Drivers of the Transition From PostCapillary Pulmonary Hypertension to Combined Pre-/PostCapillary Pulmonary Hypertension. J. Am. Heart Assoc..

[38] Fayyaz A.U., Edwards W.D., Maleszewski J.J., Konik E.A., DuBrock H.M., Borlaug B.A., Frantz R.P., Jenkins S.M., Redfield M.M. (2018). Global Pulmonary Vascular Remodeling in Pulmonary Hypertension Associated With Heart Failure and Preserved or Reduced Ejection Fraction. Circulation.

[39] Braunwald E., Braunwald N.S., Ross J., Morrow A.G. (1965). Effects of Mitral-Valve Replacement on the Pulmonary Vascular Dynamics of Patients with Pulmonary Hypertension. N. Engl. J. Med..

[40] Dalen J.E., Matloff J.M., Evans G.L., Hoppin F.G., Bhardwaj P., Harken D.E., Dexter L. (1967). Early Reduction of Pulmonary Vascular Resistance after Mitral-Valve Replacement. N. Engl. J. Med..

[41] Godino C., Salerno A., Cera M., Agricola E., Fragasso G., Rosa I., Oppizzi M., Monello A., Scotti A., Magni V. (2016). Impact and evolution of right ventricular dysfunction after successful MitraClip implantation in patients with functional mitral regurgitation. Int. J. Cardiol. Heart Vasculat..

[42] Ledwoch J., Fellner C., Hoppmann P., Thalmann R., Kossmann H., Dommasch M., Dirschinger R., Stundl A., Laugwitz K.-L., Kupatt C. (2020). Impact of transcatheter mitral valve repair using MitraClip on right ventricular remodeling. Int. J. Cardiovasc. Imaging.

[43] Hungerford S., Bart N., Jansz P., Kay S., Emmanuel S., Namasivayam M., Dahle G., Duncan A., Hayward C., Muller D.W.M. (2021). Improved right ventricular function following transapical transcatheter mitral valve implantation for severe mitral regurgitation. IJC Heart Vasc..

[44] Neuser J., Buck H.J., Oldhafer M., Sieweke J.-T., Bavendiek U., Bauersachs J., Widder J.D., Berliner D. (2022). Right Ventricular Function Improves Early After Percutaneous Mitral Valve Repair in Patients Suffering From Severe Mitral Regurgitation. Front. Cardiovasc. Med..

[45] Mandurino-Mirizzi A., Crimi G., Raineri C., Magrini G., Gazzoli F., Frassica R., Gritti V., Montalto C., Scelsi L., Turco A. (2021). Haemodynamic impact of MitraClip in patients with functional mitral regurgitation and pulmonary hypertension. Eur. J. Clin. Invest..

[46] Lim H.S., Gustafsson F. (2020). Pulmonary artery pulsatility index: physiological basis and clinical application. Eur. J. Heart Fail..

[47] Wang Z., Chesler N.C. (2011). Pulmonary vascular wall stiffness: An important contributor to the increased right ventricular afterload with pulmonary hypertension. Pulmonary Circulation..

[48] Tedford R.J. (2014). Determinants of Right Ventricular Afterload (2013 Grover Conference Series). Pulmonary Circulation..

[49] Chemla D., Lau E.M.T., Papelier Y., Attal P., Hervé P. (2015). Pulmonary vascular resistance and compliance relationship in pulmonary hypertension. Eur. Respir. J..

[50] Grant B., Lieber B. (1996). Clinical significance of pulmonary arterial input impedance. Eur. Respir. J..

[51] Wang R.-S., Huang S., Waldo S.W., Hess E., Gokhale M., Johnson S.W., Zeder K., Choudhary G., Leopold J.A., Oldham W.M. (2023). Elevated Pulmonary Arterial Compliance Is Associated with Survival in Pulmonary Hypertension: Results from a Novel Network Medicine Analysis. Am. J. Respir. Crit. Care Med..

[52] Naeije R., D’Alto M. (2016). The Diagnostic Challenge of Group 2 Pulmonary Hypertension. Prog. Cardiovasc. Dis..

[53] Linehan J.H., Haworth S.T., Nelin L.D., Krenz G.S., Dawson C.A. (1992). A simple distensible vessel model for interpreting pulmonary vascular pressure-flow curves. J. Appl. Physiol..

[54] Reeves J.T., Linehan J.H., Stenmark K.R. (2005). Distensibility of the normal human lung circulation during exercise. Am. J. Physiol.-Lung Cellul. Molecul. Physiol..

[55] Malhotra R., Dhakal B.P., Eisman A.S., Pappagianopoulos P.P., Dress A., Weiner R.B., Baggish A.L., Semigran M.J., Lewis G.D. (2016). Pulmonary Vascular Distensibility Predicts Pulmonary Hypertension Severity, Exercise Capacity, and Survival in Heart Failure. Circul. Heart Fail..

[56] Chemla D., Berthelot E., Weatherald J., Lau E.M.T., Savale L., Beurnier A., Montani D., Sitbon O., Attal P., Boulate D. (2021). The isobaric pulmonary arterial compliance in pulmonary hypertension. ERJ Open Research..

[57] Milnor W.R., Conti C.R., Lewis K.B., O'Rourke M.F. (1969). Pulmonary Arterial Pulse Wave Velocity and Impedance in Man. Circ. Res..

[58] Naeije R., Richter M.J., Rubin L.J. (2021). The physiological basis of pulmonary arterial hypertension. Eur. Respir. J..

[59] Lammers S., Scott D., Hunter K., Tan W., Shandas R., Stenmark K. (2012). Mechanics and Function of the Pulmonary Vasculature: Implications for Pulmonary Vascular Disease and Right Ventricular Function. Compr. Physiol..

[60] Naeije R., Vachiery J.-L., Yerly P., Vanderpool R. (2013). The transpulmonary pressure gradient for the diagnosis of pulmonary vascular disease. Eur. Respir. J..

[61] Bellofiore A., Chesler N.C. (2013). Methods for measuring right ventricular function and hemodynamic coupling with the pulmonary vasculature. Ann. Biomed. Eng..

[62] Lankhaar J.-W., Westerhof N., Faes T.J.C., Tji-Joong Gan C., Marques K.M., Boonstra A., van den Berg F.G., Postmus P.E., Vonk-Noordegraaf A. (2008). Pulmonary vascular resistance and compliance stay inversely related during treatment of pulmonary hypertension. Eur. Heart J..

[63] Nichols W.W., O’Rourke M.F. (1998).

[64] Saouti N., Westerhof N., Postmus P.E., Vonk-Noordegraaf A. (2010). The arterial load in pulmonary hypertension. Europ. Respirat. Rev. Off. J. Europ. Respirat. Soc..

[65] Tedford R.J., Mudd J.O., Girgis R.E., Mathai S.C., Zaiman A.L., Housten-Harris T., Boyce D., Kelemen B.W., Bacher A.C., Shah A.A. (2013). Right Ventricular Dysfunction in Systemic Sclerosis-Associated Pulmonary Arterial Hypertension. Circ. Heart Fail..

[66] Rueben S. (1971). Compliance of the Human Pulmonary Arterial System in Disease. Circ. Res..

[67] Lankhaar J.-W., Westerhof N., Faes T.J.C., Marques K.M.J., Marcus J.T., Postmus P.E., Vonk-Noordegraaf A. (2006). Quantification of right ventricular afterload in patients with and without pulmonary hypertension. Am. J. Physiol.-Heart Circulat. Physiol..

[68] Grandin E.W., Zamani P., Mazurek J.A., Troutman G.S., Birati E.Y., Vorovich E., Chirinos J.A., Tedford R.J., Margulies K.B., Atluri P. (2017). Right ventricular response to pulsatile load is associated with early right heart failure and mortality after left ventricular assist device. J. Heart Lung Transplant..

[69] Vanden Eynden F., Bové T., Chirade M.-L., Van Nooten G., Segers P. (2018). Measuring pulmonary arterial compliance: mission impossible? Insights from a novel in vivo continuous-flow based experimental model. Pulmon. Circulat..

[70] Liu Z., Brin K.P., Yin F.C. (1986). Estimation of total arterial compliance: an improved method and evaluation of current methods. Am. J. Physiol.-Heart Circulat. Physiol..

[71] Papolos A., Fan E., Wagle R.R., Foster E., Boyle A.J., Yeghiazarians Y., MacGregor J.S., Grossman W., Schiller N.B., Ganz P. (2019). Echocardiographic determination of pulmonary arterial capacitance. Int. J. Cardiovasc. Imaging.

[72] Papolos A., Tison G.H., Mayfield J., Vasti E., DeMarco T. (2021). Echocardiographic assessment of pulmonary arterial capacitance predicts mortality in pulmonary hypertension. J. Cardiol..

[73] Bhattacharya P.T., Troutman G.S., Mao F., Fox A.L., Tanna M.S., Zamani P., Grandin E.W., Menachem J.N., Birati E.Y., Chirinos J.A. (2019). Right ventricular outflow tract velocity time integral-to-pulmonary artery systolic pressure ratio: a non-invasive metric of pulmonary arterial compliance differs across the spectrum of pulmonary hypertension. Pulmon. Circulat..

[74] Collins N., Sugito S., Davies A., Boyle A., Sverdlov A., Attia J., Stewart S., Playford D., Strange G. (2022). Prevalence and survival associated with pulmonary hypertension after mitral valve replacement: National echocardiography database of Australia study. Pulmon. Circulat..

[75] Stocker T.J., Hertell H., Orban M., Braun D., Rommel K.-P., Ruf T., Ong G., Nabauer M., Deseive S., Fam N. (2021). Cardiopulmonary Hemodynamic Profile Predicts Mortality After Transcatheter Tricuspid Valve Repair in Chronic Heart Failure. J. Am. Coll. Cardiol. Intv..

